# An Innovative Approach for Management of Unfavorable Bilateral Mandibular Lingual Undercuts With Collapsible Type Complete Denture: A Case Report

**DOI:** 10.7759/cureus.63863

**Published:** 2024-07-04

**Authors:** Keerthivasan Srinivasan, Ananya Aparoopa, Lena Priya, Harini Krish, Aarthi S

**Affiliations:** 1 Department of Prosthodontics, Tagore Dental College and Hospital, Chennai, IND; 2 Department of Dentistry, Dharanidhar Medical College and Hospital, Keonijhar, IND; 3 Department of Prosthodontics, Nandha Dental College and Hospital, Erode, IND; 4 Department of Prosthodontics, Krishnadevaraya College of Dental Sciences, Bangalore, IND; 5 Department of Prosthodontics, Rathinavel Subramaniam College of Arts and Science (RVS) Dental College and Hospital, Coimbatore, IND

**Keywords:** foldable type prosthesis, hinge dentures, lingual undercuts, collapsible denture, stainless steel hinges

## Abstract

Advanced and uneven residual ridge resorption in mandibular edentulous arches leads to non-retentive and unstable dentures. The hardness of traditional heat-cured acrylic resin makes extending the denture base into bilateral lingual undercuts challenging. This can cause supporting tissue damage, pain, and ulcerations during denture insertion and removal. Although clinical challenges related to limited mouth opening were addressed by modifying the impression technique, incorporating hinges, swing lock attachments, and stainless-steel posts to form collapsible denture bases, there are no documented case reports with proper follow-up regarding the use of such type dentures in cases of mandibular lingual undercuts. A 68-year-old male patient reported, with the chief complaint of missing teeth in the upper and lower jaws for five years and wanting replacement. The intraoral clinical examination yielded findings of a severely compromised mandibular ridge (ACP Class IV) and a moderately compromised maxillary ridge (ACP Class II). In the maxillary arch, the presence of anterior labial undercut, and bilateral undercuts lateral to tuberosity were evident. The patient reported pain on palpation bilaterally in the tuberosity region. Prolonged mandibular edentulism and uneven bone resorption resulted in unfavorable bilateral lingual undercuts, with class III (M.M. House) border tissue attachment in the labial and buccal aspects of the basal tissue area. After enumerating the treatment options, the patient opted for a removable prosthesis for the maxillary and mandibular arch. Pre-prosthetic surgery was done to eliminate tuberosity undercuts. Since the patient was unwilling to take up pre-prosthetic surgical corrections for the mandibular lingual undercuts, a significant challenge emerged: creating a retentive mandibular complete denture without compromising the peripheral seal and retention. A conventional complete denture was fabricated after blocking the unfavorable undercut and reducing the height of the flange. On the recall appointment, the patient complained of reduced retention and food lodgment in the intaglio surface of the denture and pain due to denture movement on mastication. In this case report, stainless steel hinges have been added to the lingual flange of the mandibular complete denture to make it collapsible. The resultant denture facilitated reduced tissue trauma and discomfort during denture removal and insertion and had satisfactory retention and stability compared to the former denture. These collapsible type dentures can be used as an alternative to flexible dentures, wherein patients can’t afford surgeries or flexible dentures.

## Introduction

Advanced and uneven residual ridge resorption in mandibular edentulous arches often poses the necessity to extend lingual flanges of the denture to engage the sublingual undercut [1-4]. The rigidity inherent in conventional heat-cured acrylic resin presents difficulties such as discomfort and ulcerations when attempting to extend the denture base into bilateral disto-lingual undercuts [5]. The management includes modifying the denture-bearing region by pre-prosthetic surgeries [6], adjustment of the denture base and modifying the path of insertion [7], relining materials, and flexible denture base materials.

Disadvantages such as the impracticality of pre-prosthetic surgeries diminished peripheral seal and stability arising from denture modification [8], phthalate toxicity, plasticizer leachability [9], deterioration of physical properties, and cytotoxic effects associated with relining materials [10-13], uneven force distribution, cost inefficiency, and the need for specialized diamond points for finishing flexible dentures are additional challenges [14]. Cases with limited mouth opening addressed by incorporating modifications in the impression technique, such as hinges [15], swing lock attachments [16], and stainless steel posts [17], lack proper documentation of follow-up.

In this case report, stock stainless steel hinges were incorporated into the lingual flange. This innovative addition serves to make the denture collapsible, thereby minimizing tissue trauma and discomfort during both removal and insertion. Importantly, it does not compromise the denture's retention and stability.

## Case presentation

**Figure 1:**
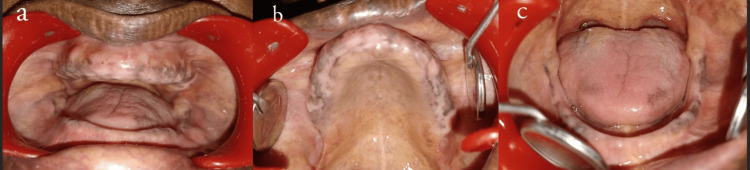
(a) Pre-operative intraoral images of maxilla and mandible. (b) Pre-operative image of maxilla showing anterior labial undercut and bilateral tuberosity undercuts. (c) Resorbed mandibular ridge with class III border tissue attachment

A 68-year-old male patient reported, with the chief complaint of missing teeth in the upper and lower jaw for five years and wanting replacement. The intraoral clinical examination yielded findings of moderately compromised maxillary and mandibular ridges. In the maxillary arch, the presence of anterior labial undercut and bilateral undercuts lateral to tuberosity were evident (Figures 1a, 1b). The patient reported pain on palpation bilaterally in the tuberosity region. Prolonged mandibular edentulism and uneven bone resorption resulted in unfavorable bilateral lingual undercuts, with class III (M.M. House) border tissue attachment in the labial and buccal aspects of the basal tissue area (Figure 1c).

Various treatment options, including implant-supported over-dentures, pre-prosthetic surgery, and flexible complete dentures, were discussed with the patient. The patient chose a cost-effective solution, opting for a removable complete denture for the maxillary arch after undergoing pre-prosthetic surgery to address unilateral lateral exostosis. Despite being unwilling to undergo pre-prosthetic surgical corrections for the mandibular lingual undercuts, a significant challenge is creating a retentive mandibular complete denture without compromising the peripheral seal and retention. Denture fabrication was done conventionally till the wax trial. Before denture processing, the cast was modified by blocking lingual undercuts [8]. The patient was dissatisfied with the resultant denture due to food lodgment, instability, and pain due to denture movement on mastication.

Since the patient denied a flexible denture concerning the cost factor, an alternate treatment plan involved the fabrication of a collapsible denture in the mandible without altering the denture base retentive areas [7,14]. The patient consented to the trial of the procedure.

**Figure 2:**
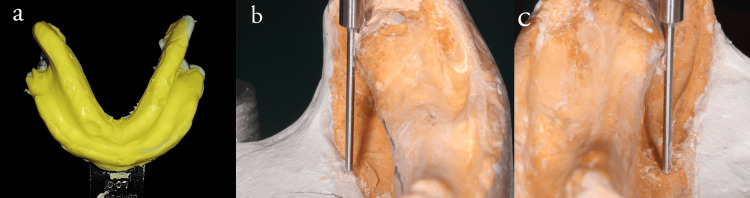
(a) Primary impression made using stock tray with irreversible hydrocolloid material after pre-molding the tray borders with elastomer (b & c) left and right disto-lingual sulcus undercuts are evaluated with a surveyor

Conventional steps for fabricating a complete denture were carried out in the maxilla till the wax-up procedure. For the mandible, a preliminary impression was made using a stock tray with irreversible hydrocolloid (Figure 2a). The lingual flange extension was pre-molded using putty elastomer. On the primary cast, the right and left disto-lingual undercuts are evaluated with a surveyor (Figures 2b, 2c). Custom tray fabrication is done with auto-polymerizing resin (DPI-RR cold cure) on the primary cast after surveying and blocking out unfavorable lingual undercuts.

**Figure 3:**
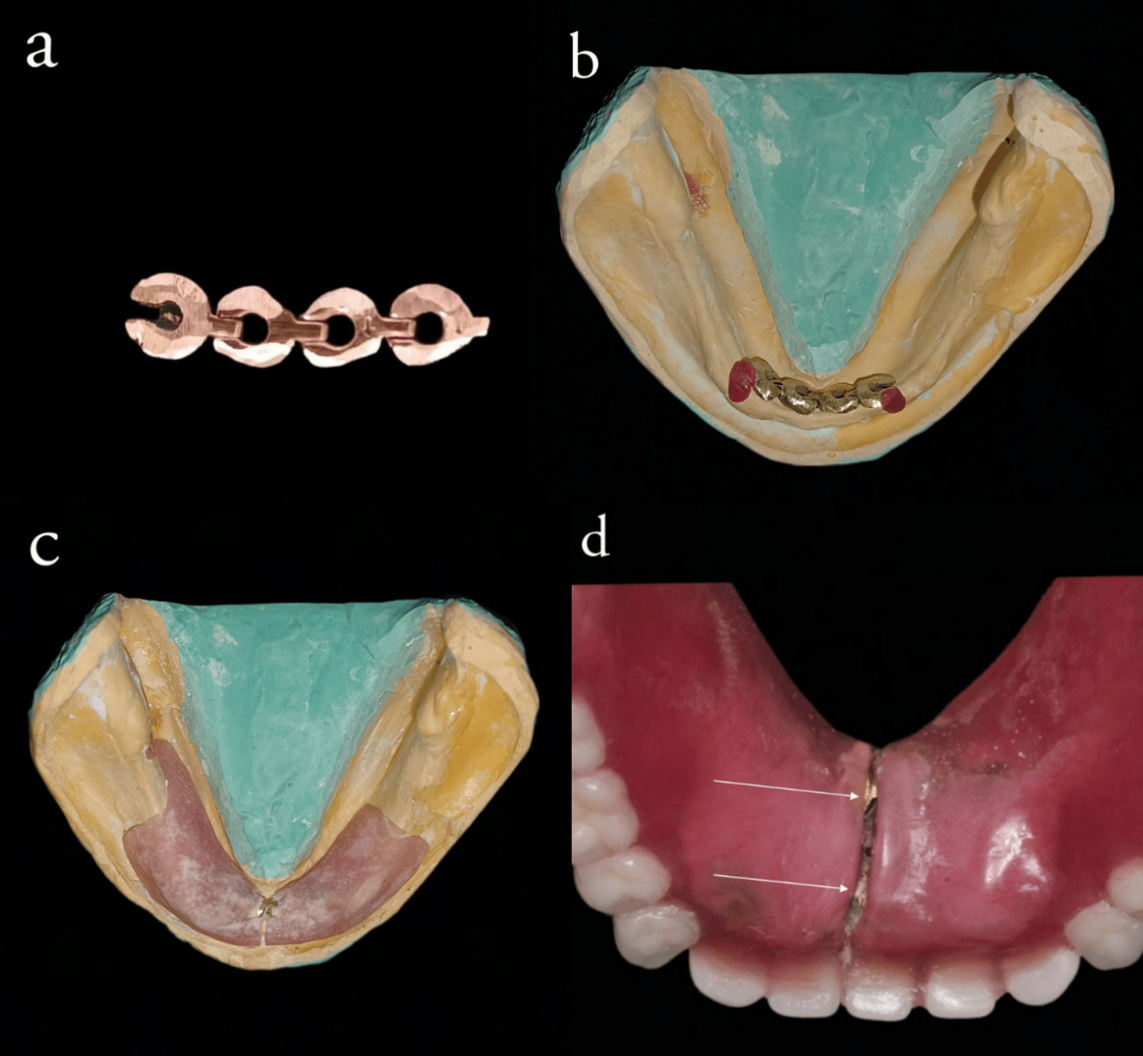
a) stock stainless steel hinges. (b) The extent of hinge connections is checked on the duplicated definitive cast, and it is stabilized with heat-cured denture base resin. (c) Heat cure record base is fabricated by means of compression molding technique with the hinge stabilized with heat cure acrylic resin and sectioned in the midline corresponding to the lingual notch area. (d) After verifying the anterior occlusal rim height with a tentative jaw relation record for adequate space, the second stainless steel hinge was incorporated lingually, corresponding to the mandibular central incisor region 3 mm superior to the first hinge.

Sectional border molding and the final impression made with the putty wash technique. Record base adaptation and occlusal rim fabricated on the definitive cast after blocking unfavorable undercuts. Tentative jaw relation recorded, articulation carried out with mean value articulator (AGSON). Stock stainless steel hinge (Figure 3a) is placed on the lingual notch area, and the extent of hinge connections is checked on the duplicated definitive cast, which is stabilized with heat-cured denture base resin (Figure 3b). The mandibular permanent record base was fabricated by means of the compression molding technique, along with the hinge stabilized with heat-cured acrylic resin. It was sectioned in the midline corresponding to the lingual notch area (Figure 3c). After verifying the anterior occlusal rim height with a tentative jaw relation record for adequate space, the second stainless steel hinge was incorporated lingually, corresponding to the mandibular central incisor region 3 mm superior to the first hinge in the above-mentioned manner (Figures 3b, 3d).

**Figure 4:**
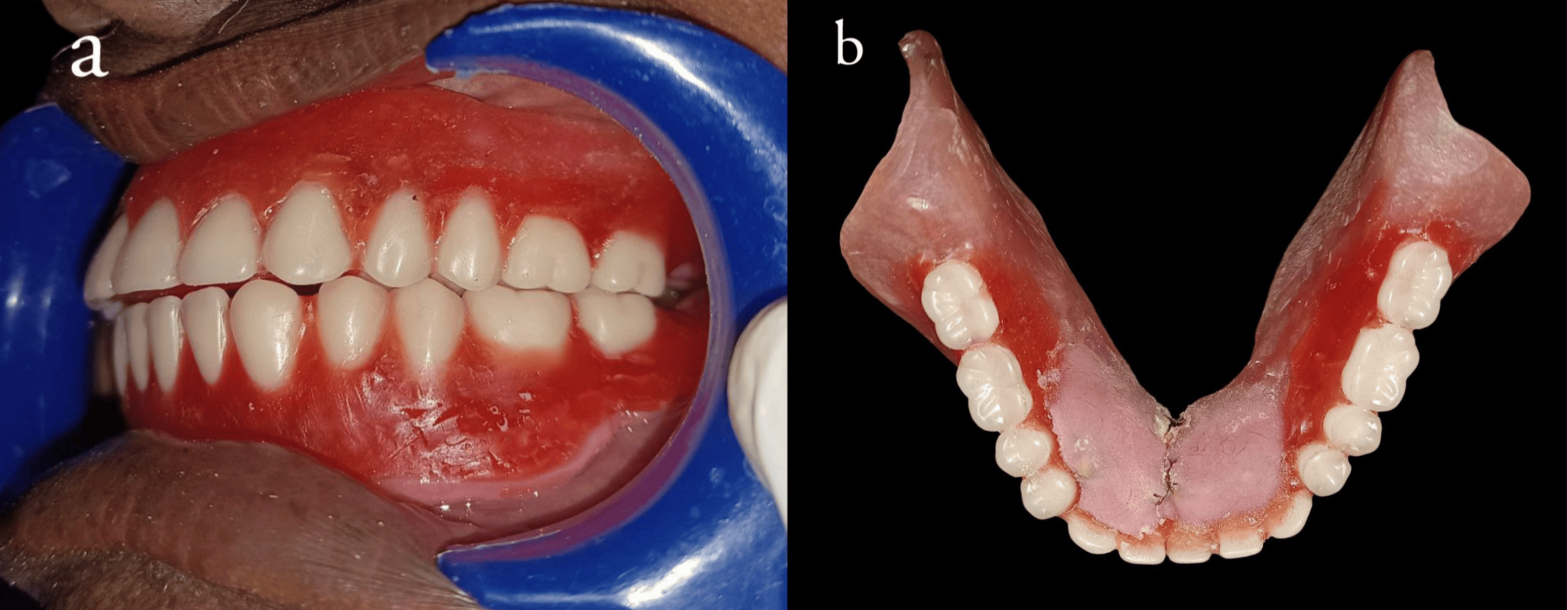
(a) Teeth arrangement and wax try-in. (b) The second hinge is placed after evaluating the height of the lower anterior occlusal plane during the wax try-in procedure

The hinge-incorporated base is transferred to the articulated master cast, and tooth arrangement is done. A wax trial with a collapsible trial denture was checked for occlusion, stability, aesthetics, and patient comfort (Figure 4a). After evaluating the height of the lower anterior occlusal plane, a second hinge is incorporated (Figure 4b).

**Figure 5:**
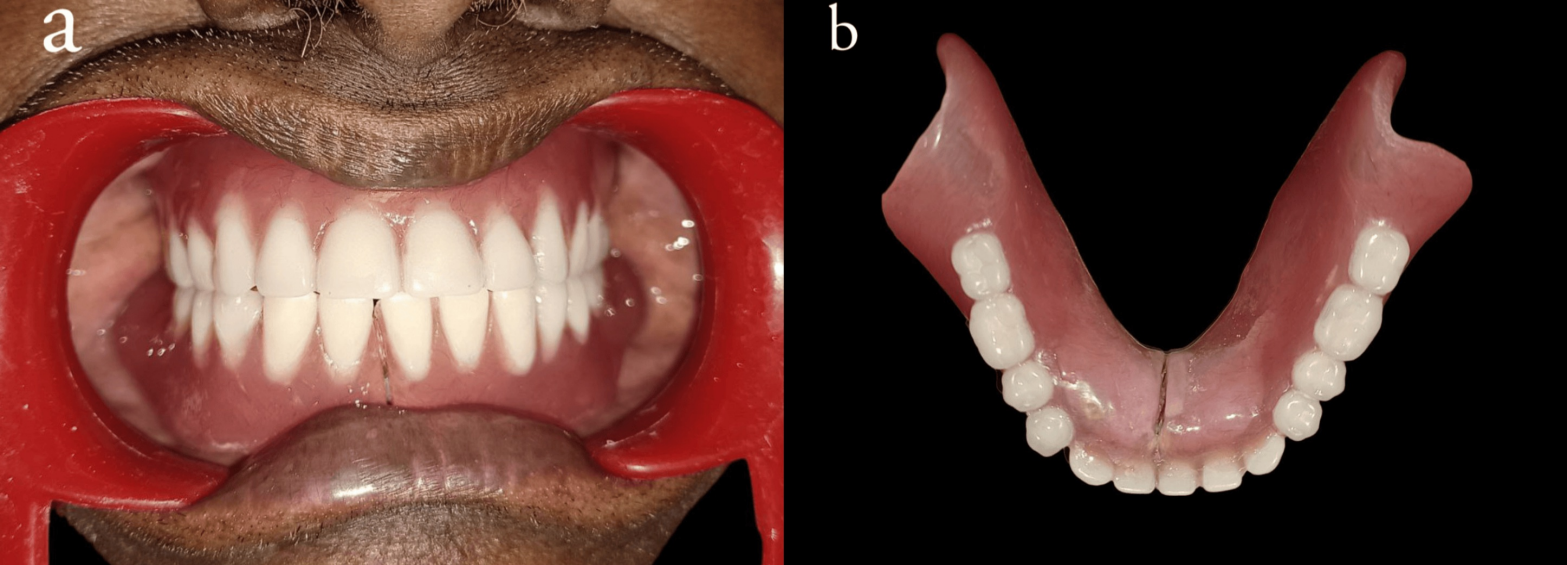
(a) Denture insertion (b) Finished and polished denture

A permanent soft liner (DETAX MOLLOSIL) was used to reline the intaglio surface during denture insertion after finishing and polishing (Figure 5a, 5b) to facilitate improved tissue adaptation.

**Figure 6:**
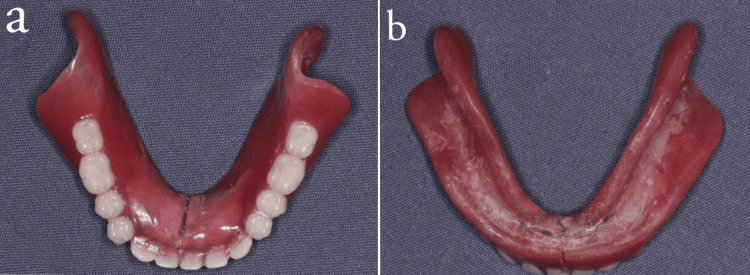
(a, b) occlusal surface and intaglio surface and of denture after six months of follow-up

Clinical examination after a six-month follow-up revealed no cytotoxic changes in the oral mucosa, as well as the surface of the stainless steel link, showed no appreciable deteriorative changes with satisfactory occlusal and intaglio surfaces (Figure 6a, 6b), and the prosthesis had satisfactory stability with no food lodgment beneath the prosthesis. 

## Discussion

In this case report, the patient had a moderately compromised maxillary ridge (Type C), and the mandible had a residual bone height of 11 mm at the least vertical height of the mandible (Type III). The location of the attached mucosal base was present in all the regions except anterior buccal and lingual vestibules (Type C) (Figure 1a). The maxillomandibular relation was found to be class I, with a need for pre-prosthetic surgery in the maxilla and mandible for unfavorable tuberosity exostosis and unfavorable lingual undercuts. Therefore, the patient was categorized under class III of the ACP classification. 

According to previous literature [18], patients with mandibular bilateral lingual undercuts are managed by blocking the unfavorable undercuts on the cast and by altering the height of the denture flange to the level of the crest of the undercut. In this case, a conventional complete denture was fabricated after blocking the unfavorable undercut and reducing the height of the flange. On the recall appointment, the patient complained of reduced retention and food lodgment in the intaglio surface of the denture. When examined, there was a diminished peripheral seal, which was due to blockage of the undercut and a reduction in the flange height.

Previous literature with limited mouth opening was addressed by incorporating certain modifications in the impression technique, with the use of sectional custom trays connected by the components such as locking levers, hinges, orthodontic expansion screws, and the resulting collapsible dentures were connected by hinges [19], swing lock attachments [20], etc. Also, previous studies have provided descriptions of collapsible dentures that incorporated a flexible silicone union to join the two sides of the dentures [21]. These modifications facilitated easy insertion and removal of the prosthesis since conventional non-collapsible types of complete dentures would be larger in patients with reduced circumoral width. As per the literature, the success of the modified forms of oral prosthesis depended on the extent of the microstomia and the nature of the cause of the microstomia. The common limitation of the above-mentioned innovations included a lack of documentation in follow-ups, so the long-term success rate of the prosthesis was not mentioned in any of the literature.

To date, no case reports have been published incorporating these types of hinge connections in complete dentures for patients with unfavorable mandibular lingual undercuts. In the present case, to overcome the complaints of the patient, the above-mentioned innovative hinge mechanism was incorporated into the denture. Further, to prevent the supero-inferior movement and to restrict the mediolateral movement of the denture, two hinges were incorporated lingually, corresponding to the mandibular central incisor region, with 3 mm space between each connection.

Before insertion, the mandibular prosthesis is folded medially toward the tongue. To fit onto the lingual undercuts and rest on the alveolar ridge, the prosthesis is stretched laterally [22]. The acrylic resin denture teeth and hinge mechanism prevent occlusal rocking. They also enable prosthetics that fully extend the lingual flange, even with substantial bilateral lingual undercuts, improving patient comfort and satisfaction over the previous denture.

Since there is no mechanism to secure the denture in an extended position, tongue volume and control are essential to maintaining prosthetic function and shape. The patient's larger tongue size, effective psychomotor skills, and well-shaped polished denture surface helped maintain the spread-out denture position and improve stability and retention. To avoid tissue entrapment during hinge mechanism action, the soft tissue underneath the hinge assembly was relieved. The double hinge connection used in this case facilitated ease in insertion and removal without any tissue trauma. Since the lingual flanges mechanically engage both the undercuts, the stability of the prosthesis was significantly improved during mastication and other functional movements, which was qualitatively assessed [23]. As the denture flange extension, denture base adaptation to the underlying basal seat area, and mechanical engagement of the denture in the retentive undercuts were achieved, the retention of the prosthesis was satisfactory. The food lodgment that the patient complained about was also resolved since no alterations were made to the flange extension.

In this case report, follow-ups were done periodically till six months, and the basal seat tissues showed no deteriorative clinical changes. The prosthesis showed satisfactory occlusal and intaglio surface (Figure 6a, 6b). The long-term success rate of the prosthesis has to be assessed. 

## Conclusions

This hinge-type collapsible denture showed better stability due to the mechanical engagement of bilateral undercuts when compared to the conventional non-collapsible prosthesis in which critical flange areas are relieved. These collapsible dentures can be used to manage cases with severe lingual undercuts effectively wherein patients can’t afford pre-prosthetic surgeries. This approach exhibits the drawbacks that are inherent to all foldable prostheses, including the need for extra time and lab support. However, to ascertain the enduring success of this strategy, it is necessary for periodic recall maintenance and to make further improvements in design to maintain the spread-out configuration of the prosthesis.
